# Comparison of DNA-Hydrolyzing Antibodies from the Cerebrospinal Fluid and Serum of Patients with Multiple Sclerosis

**DOI:** 10.1371/journal.pone.0093001

**Published:** 2014-04-15

**Authors:** Taisiya A. Parkhomenko, Vasilii B. Doronin, Massimiliano Castellazzi, Marina Padroni, Michela Pastore, Valentina N. Buneva, Enrico Granieri, Georgy A. Nevinsky

**Affiliations:** 1 Institute of Chemical Biology and Fundamental Medicine, Russian Academy of Sciences, Siberian Division, Novosibirsk, Russia; 2 Novosibirsk Medical University, Ministry of Public Health of Russian Federation, Novosibirsk, Russia; 3 Multiple Sclerosis Center, Department of Neurology, Ferrara University, Ferrara, Italy; 4 Novosibirsk State University, Novosibirsk, Russia; University of Missouri-Kansas City, United States of America

## Abstract

It was found that high-affinity anti-DNA antibodies were one of the major components of the intrathecal IgG response in multiple sclerosis (MS) patients [Williamson et al., PNAS, 2001]. Recently we have shown that IgGs from the sera of MS patients are active in the hydrolysis of DNA. Here we have shown, for the first time, that average concentration of total proteins (132-fold), total IgGs (194-fold) and anti-DNA antibodies (200-fold) in the sera is significantly higher than that in the cerebrospinal fluid (CSF) of fifteen MS patients. The relative activities of total protein from sera and CSFs varied remarkably from patient to patient. It was surprising that the specific DNase activity of the total protein of CSF reparations were 198-fold higher than the serum ones. Electrophoretically and immunologically homogeneous IgGs were obtained by sequential affinity chromatography of the CSF proteins on protein G-Sepharose and FPLC gel filtration. We present first evidence showing that IgGs from CSF not only bind but efficiently hydrolyze DNA and that average specific DNase activity of homogeneous antibodies from CSF is unpredictably ∼49-fold higher than that from the sera of the same MS patients. Some possible reasons of these findings are discussed. We suggest that DNase IgGs of CSF may promote important neuropathologic mechanisms in this chronic inflammatory disorder and MS pathogenesis development.

## Introduction

Multiple sclerosis (MS) is a chronic demyelinating pathology of the central nervous system presenting a serious medical and social problem. Its etiology remains unclear, and the most valid theory of its pathogenesis assigns the main role in the destruction of myelin-proteolipid shell of axons to inflammation related with autoimmune reactions ([1] and refs therein). Although the T-cell immune system plays a leading role in MS pathogenesis, normal functioning of the B-cell system is also important for the disease development. An enhanced synthesis of immunoglobulins (usually IgGs), their free light chains, and of polyspecific DNA binding antibodies (Abs) interacting with phospholipids are observed in MS patients [1].

New keys for understanding MS pathogenesis have appeared after cloning IgG repertoire directly from active plaques and periplaque regions in MS brains and from B-cells recovered from the cerebrospinal fluid of a patient with MS with a subacute disease [2]. It was found that high affinity anti-DNA Abs were a major component of the intrathecal IgG response in MS patients. Furthermore, DNA-specific monoclonal Abs derived from two MS individuals as well as a DNA-specific Ab derived from a systemic lupus erythematosus (SLE) patient bound efficiently to the surface of neuronal cells and oligodendrocytes. For these Abs, cell-surface recognition was DNA-dependent. The findings indicate that anti-DNA Abs may promote important neuropathologic mechanisms in chronic inflammatory disorders, such as MS and SLE [2].

Artificial abzymes (catalytic antibodies against transition state analogues of chemical reactions) and natural abzymes are novel biological catalysts that have attracted much interest in the last years (reviewed in [3]–[7]). Artificial abzymes are abzymes against analogs of transition states of catalytic reactions [3]–[7] or antiidiotypic Abs induced by a primary antigen, which may show some of its features including the catalytic activity (for review also see [8]–[12]). During past two decades it has become clear that auto-antibodies (auto-Abs) from the sera of patients with different autoimmune diseases can possess enzymatic activities and that their occurrence is a distinctive feature of autoimmune diseases (reviewed in [13]–[17]). It is thought that abzymes may play a significant role in forming specific pathogenic patterns and clinical settings in different autoimmune conditions through their broadened auto-Ab properties [13]–[17]. Patients with autoimmune diseases produce Abs to nucleoprotein complexes, to DNA and to enzymes that participate in nucleic acid metabolism [13]–[17]. In autoimmune diseases, there can be a spontaneous induction of anti-idiotypic antibodies, which are Abs elicited by a primary antigen, including some with catalytic activity, or transition from polyreactive catalytic activity to autoantigen-directed activity. Natural abzymes hydrolyzing DNA, RNA, polysaccharides, oligopeptides, and proteins are present in the serum of patients with several autoimmune and viral diseases (reviewed in [13]–[17]). Healthy humans do not develop abzymes with detectable DNase and RNase activities, their levels being usually on the borderline of sensitivity of the detection methods [13]–[17].

It has recently been shown that myelin basic protein (MBP)-hydrolyzing activity is an intrinsic property of IgGs, IgMs, and IgAs from the sera of MS patients [17]–[22]. Recognition and degradation of MBP peptides by serum auto-Abs were confirmed as a novel biomarker for MS [21]–[22]. The established MS drug Copaxone appears to be a specific inhibitor of MBP-hydrolyzing abzyme activity [22].

At the same time, as mentioned above, anti-DNA Abs were found to be a major component of the intrathecal IgG response in MS patients [2]. First DNase abzymes were found in the sera of SLE patients [23]. Then, it was shown that IgGs and IgMs from the sera of MS patients effectively hydrolyzed DNA, RNA, and oligosaccharides [24]–[28]. Whereas only 18 and 53% of MS patients contained increased concentrations of Abs to native and denatured DNA, respectively, as compared with healthy donors, DNase abzymes were found in ∼80–90% of MS patients [17], [24], [25]. Since DNase abzymes of MS patients [14] similarly to SLE patients [28] are cytotoxic, cause nuclear DNA fragmentation and induce cell death by apoptosis, they can play an important role in SLE and MS pathogenesis. Taking these observations into account, analysis of relative concentrations of proteins, canonical enzymes and DNase abzymes in the cerebrospinal fluid (CSF) of MS patients is of special interest.

In the present study we have used different approaches to provide, for the first time, a very strong direct evidence that DNase activity is intrinsic to IgGs from CSF of MS patients and compared some other parameters characterizing the MS CSF and sera.

## Results

Fifteen patients (11 women and 4 men) satisfying the criteria for clinically or laboratory-supported definite MS according to [29], [30] were retrospectively selected for the study. Of these, 13 were relapsing–remitting (RR), and 2 were primary progressive (PP) in agreement with the criteria of Lublin and Reingold [31]. Clinical course (RR and PP), clinical activity (relapse at time of sampling), and MRI activity (the presence of gadolinium enhancing lesions at MRI examination) were analyzed as described previously [32]. The characteristics of the MS patients are summarized in Table 1.

**Table 1:** Several different characteristics of MS patients.

Number of patient	Sex	Age, years	Clinical course*	Clinical activity**	MRI activityξ
1	male	59	PP	yes	yes
2	female	28	RR	no	no
3	female	36	RR	yes	yes
4	male	26	RR	yes	no
5	male	49	RR	no	no
6	female	20	RR	yes	no
7	female	46	PP	yes	no
8	female	51	RR	yes	yes
9	female	31	RR	yes	no
10	female	26	RR	no	no
11	female	43	RR	yes	yes
12	male	45	RR	yes	no
13	female	30	RR	no	yes
14	female	60	RR	yes	no
15	female	34	RR	yes	yes

*Relapsing–remitting (RR) and primary progressive (PP) MS.

**Clinical activity = presence of relapse at the time of sampling.

ξ MRI activity = presence or absence gadolinium enhancing lesions at MRI examination.

It was interesting to compare some different indexes for CSF and sera of MS patients. Therefore, first we measured a relative concentration of total protein in CSF and sera of MS patients (Table 2). The relative concentrations of total protein of CSFs (range 0.26–0.66 mg/ml) and sera (47–74 mg/ml) of fifteen MS patients varied in different ranges. The average concentration of the total protein in the serum (62.5±6.7 mg/ml) was ∼130-fold higher compared with CSF (0.48±0.09 mg/ml) and these values did not demonstrate good correlation (coefficient correlation (CC) = −0.12), Table 2). The relative concentrations of total IgGs in the serum and CSF were first measured using an immunoblotting test system. The relative concentration of total IgGs in the serum (range 7.9–16.6 mg/ml; average value 11.7±1.8 mg/ml) was 167-fold higher than that for the CSF (range 0.02–0.19 mg/ml; average value 0.07±0.04 mg/ml) and there was not good correlation between these values, CC = +0.07.

**Table 2:** The relative concentration of total protein, IgGs, and anti-DNA antibodies in CSF and sera of patients with MS.*

Num. of patient	Relative concentration of total proteins, mg/ml		Relative concentration of total IgGs, mg/ml		Relative concentration of total anti-DNA Abs, A_450_ ξ	
	CSF (1)	Serum (2)	CSF (3)	Serum (4)	CSF (5)	Serum (6)
1	0.55	59	0.11	11.9	0.22	110
2	0.33	63	0.19	9.3	0.24	2000
3	0.26	72	0.03	12.8	0.21	300
4	0.51	63	0.06	11.6	0.21	160
5	0.64	48	0.06	10.6	0.22	110
6	0.58	64	0.05	11.4	0.22	290
7	0.56	64	0.11	10.8	0.22	430
8	0.66	74	0.14	14.5	0.23	320
9	0.37	73	0.08	16.6	0.21	210
10	0.39	60	0.03	10.1	0.23	370
11	0.42	58	0.02	14.9	0.21	130
12	0.53	77	0.05	10.0	0.24	260
13	0.39	57	0.03	10.0	0.23	850
14	0.47	58	0.09	12.6	0.22	790
15	0.48	47	0.02	7.9	0.22	220
Average values**	0.48±0.09	62.5±6.7	0.07±0.04	11.7±1.8	0.22±0.008	437±311
Ratio of the values	130		167		1986	
Coefficient correlation	−0.12 (p<0.05)		+0.074 (p<0.05)		+0.57 (p<0.05)	
Coefficients of correlation between different values corresponding to numbers of this Table columns						
Numbers	1 and 3	1 and 5	2 and 4	2 and 6	3 and 5	4 and 6
Coefficient correlation	+0.17 (p<0.05)	+0.11 (p<0.05)	+0.47 (p<0.05)	−0.004 (p<0.05)	+0.4 (p<0.05)	+0.32 (p<0.05)

*For each value, a mean of three measurements is reported; the error of the determination of values did not exceed 7–10%.

**Average values are reported as mean ± S.E.

ξ Different dilution CSF (2-fold) and serum (1000-fold) preparations were used; relative content was recalculated for initial undiluted CSFs and sera.

IgGs from the serum and CSF were purified by affinity chromatography on protein-G Sepharose and IgG-protein concentration was estimated in the peaks eluted from the sorbent by an acidic buffer (see below). The relative concentration of total IgGs determined by two different methods was the same within the experimental error. Interestingly, the concentration of total protein in CSF was 6.9-fold higher than total IgGs, while this difference in the case of the serum (5.4-fold) was by a factor of 1.3 lower.

We estimated the relative concentration of anti-DNA antibodies in CSF and serum. The relative concentration of anti-DNA antibodies in the CSF of all patients was measured after dilution of CSFs 2-fold; the values for all CSFs were comparable (range 0.21–0.24 A_450_ units counting on a cerebrospinal fluid without dilution; average value 0.22±0.008 A_450_ units), while in the case of the serum (dilution 1000-fold) it was significantly varied (range 110–2000 A_450_ units counting on serum without dilution; average value 437±311 A_450_ units). Since the relative concentration of anti-DNA antibodies in CSF was relatively low, we have removed all Abs from CSFs by passage through two columns: protein G-Sepharose and protein A-Sepharose. CSFs passed through the columns and controls containing only a standard buffer demonstrated the same A_450_ values, which were ≤5% in comparison with those for untreated CSFs. Thus, we have shown that the relatively low A_450_ values corresponding to untreated CSFs are caused by anti-DNA antibodies in these CSFs. In addition, we have shown that DNA-hydrolyzing activity is an intrinsic property of IgGs from CSF (see below).

There was no good correlation between anti-DNA antibodies in the sera and CSFs, CC = +0.57 (Table 2). In addition, the correlation was very low between the total concentration of IgGs and anti-DNA antibodies both in CSF (CC = +0.40) and the serum (CC = −0.32). At the same time, the average differences in the concentration of total IgGs for the serum and the CSF (167-fold) were 11.9-fold lower than the difference for anti-DNA antibodies of the serum and the CSF (1986-fold) (Table 2).

**Figure 1:**
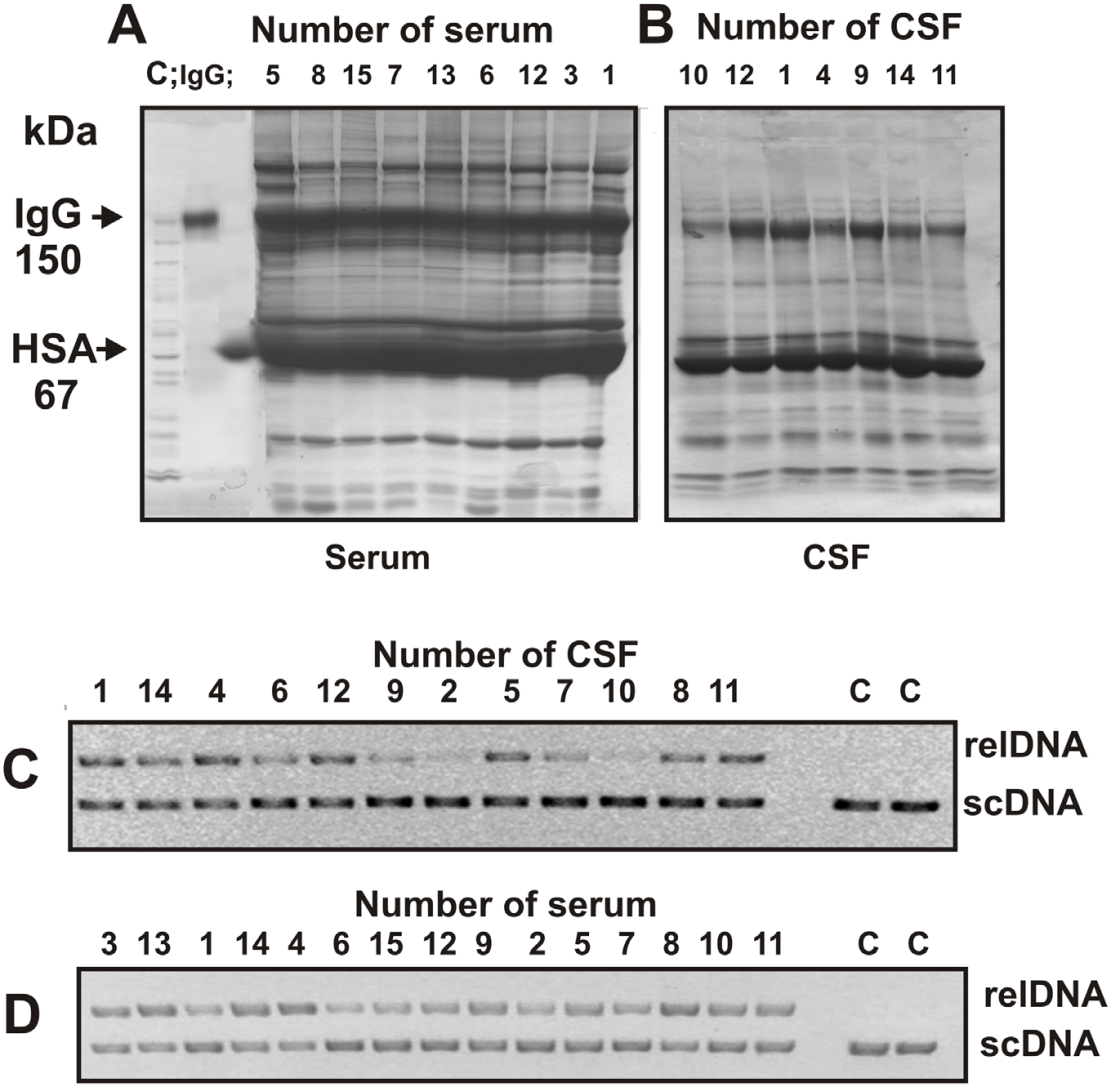
SDS-PAGE analysis of total proteins corresponding to the sera (40 µg) (A) and CSFs (30 µg) (B) of several MS patient in a nonreducing 3–16% gradient gel followed by Coomassie staining. The arrows (lane C) indicate the positions of molecular mass markers. The relative DNase activity of total protein preparations corresponding to the serum (C) and CSF (D) of several patients. Reaction mixtures were incubated for 2 h at 37°C and contain initial protein preparations diluted finally 1000- and 15-fold in the case of serum and CSF preparations, respectively; Lanes C (C and D) correspond and scDNA incubated without Abs.

It is known that human sera contain several major and especially minor proteins. The average concentration of the total protein in the serum was ∼130-fold higher than in CSF (Table 2). It was interesting to compare major proteins of the sera and CSFs using SDS polyacrylamide gel electrophoresis (SDS-PAGE). Fig. 1 demonstrates major proteins in some samples of the sera and the CSFs. One can see that the sera contain more major proteins, but in both cases IgGs (150 kDa) and human serum albumin (∼67 kDa) are most major proteins. At the same time, in contrast to CSFs, the sera contain several major proteins with mol. masses higher than 150 kDa as well as lower than 150 kDa, but higher than 67 kDa (Fig. 1). It is interesting to see that in CSFs there are several additional good visible proteins with electrophoretic mobility lower than 67 kDa in comparison with serum preparations.

It was interesting to compare total DNase relative activity (RA) of all DNA-hydrolyzing enzymes and Abs in the sera and CSF of MS patients. Preparations of total protein were capable to hydrolyze supercoiled plasmid DNA (scDNA), forming single breaks in one strand of supercoiled DNA (relaxed DNA) and then multiple breaks yielding linear DNA. Finally, they hydrolyzed DNA into short and medium-length oligonucleotides. However, after such a deep hydrolysis of DNA, it was very difficult to estimate the relative activity of these preparations. The RAs of total protein from sera and CSF varied remarkably from patient to patient. Fig. 1 illustrates typical examples of cleavage of scDNA by total protein from several MS patients after 2 h of incubation. Therefore, in order to estimate the DNase activity quantitatively, we found the concentration for each preparation and the time of incubation sufficient to convert scDNA into the relaxed form (10–40%; for example, lanes 2, 6, 7, 9, and 10 of Fig. 1C) without further noticeable fragmentation after 1–2 h of incubation. Since all measurements (initial rates) were taken within the linear regions of the time courses and protein concentration, the measured RAs were normalized to standard conditions (pmole DNA/1 mg of protein/1 h; standard units (SU)) (Table 3).

**Table 3:** Relative DNase activity of total proteins and IgGs from CSF and sera of patients with MS.*.

Number of patient	Relative specific DNase activity of total proteins; pmole DNA/1 mg of protein/1 h			Relative DNase activity of total proteins; pmole DNA/1 ml of initial solution/1 h			Relative specific DNase activity of IgGs; pmole DNA/1 mg of Ab/1 h		
	CSF (7)	Serum (8)		CSF (9)		Serum (10)	CSF (11)		Serum (12)
1	118	0.41		64		24	528.9		17.5
2	70	0.38		23		24	716		5.2
3	263	0.33		69		23	778.9		3.6
4	118	0.43		62		27	nd		ndµ
5	25	0.72		16		34	564.6		10.5
6	62	0.26		36		17	nd		nd
7	55	0.43		31		28	544.2		13.1
8	25	0.46		14		34	129.3		17.6
9	78	0.47		29		34	117.3		17,0
10	44	0.75		17		45	210.9		7.8
11	133	0.61		56		35	1190.5		20.8
12	100	0.33		53		25	928.6		12.2
13	244	0.76		95		44	171.8		3.9
14	78	0.88		51		51	629.3		11.6
15	100	0.43		44		20	552.7		10.4
Average value**	101±50	0.51±0.16		41.3±18.8		31±8	543.3±239.7		11.2±4.3
Ratio of the values	198.0			1.3			48.5		
Coefficient correlation	−0.05 (p<0.05)			0.03 (p<0.05)			0.26 (p<0.05)		
Coefficients of correlation between different values corresponding to column numbers of Tables 2 and 3									
Numbers	1 and 7		2 and 8	3 and 11	4 and 12			5 and 11	6 and 2
Coefficient correlation	+0.61 (p<0.05)		−0.49 (p<0.05)	+0.16 (p<0.05)	+0.58 (p<0.05)			+0.11 (p<0.05)	+0.51 (p<0.05)

*For each value, a mean of three measurements is reported; the error of the determination of values did not exceed 7–10%.

**Average values are reported as mean ± S.E.

µ No determined.

The relative specific DNase activity of total protein of the serum preparations was varied in the range 0.26–0.88 pmole DNA/1 mg of protein/1 h (average value 0.51±0.16 SU). It was surprising that the specific activity of the total protein of CSF reparations were 198-fold higher (range 25–263 SU, average value 101±50 SU) than the serum ones (Table 3). At the same time, concentration of total protein in the case of CSF was 130-fold lower than that of sera (Table 2). Therefore, in calculation on 1 ml of liquid the average RA of total protein corresponding to the sera was only 1.5-fold lower than that for CSFs.

**Figure 2:**
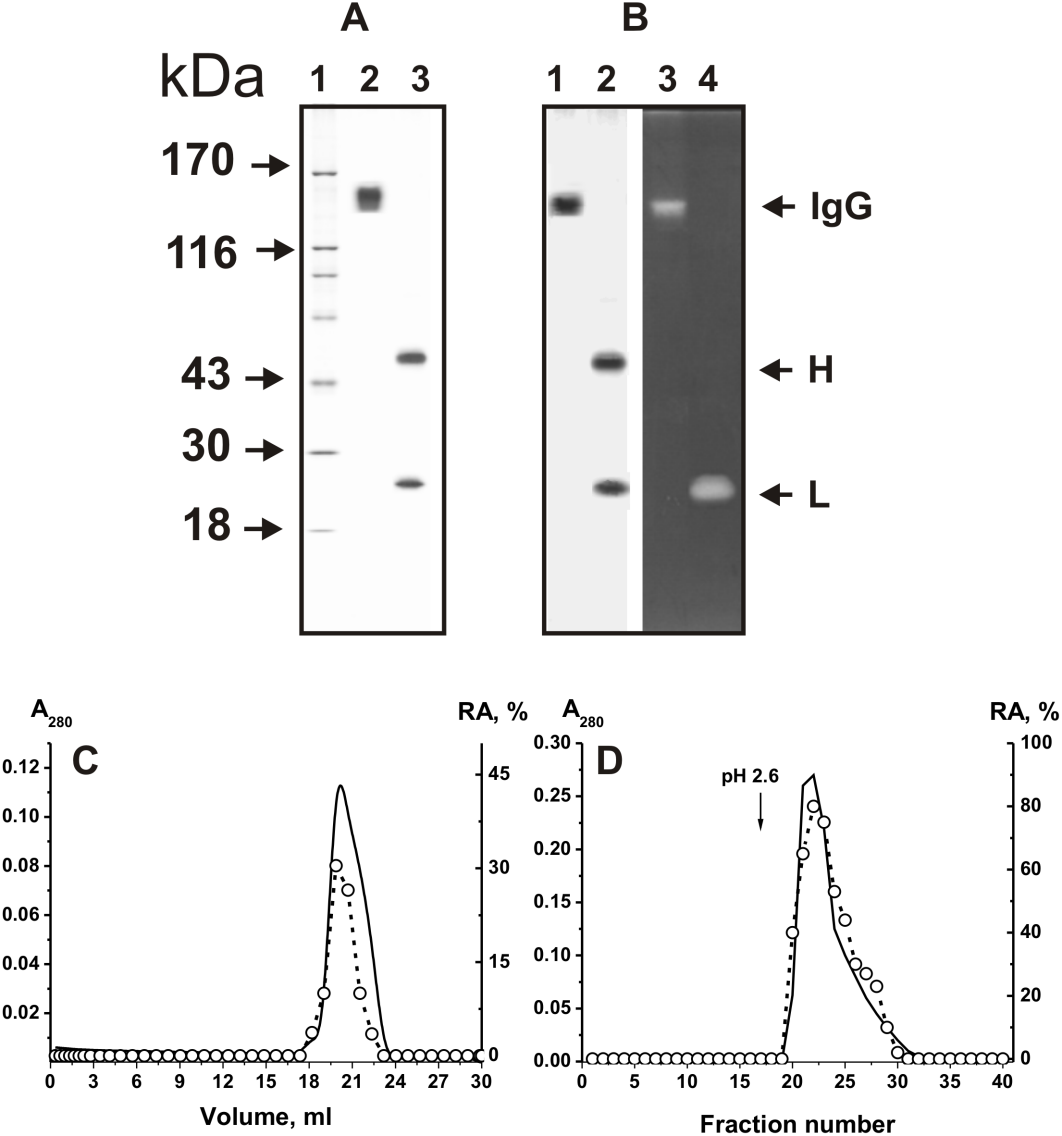
SDS-PAGE analysis of IgG_mix_ (7 µg) corresponding to 13 CSFs of MS patients in 3–16% gradient gel before (lane 2) and after treatment with DTT (lane 3) followed by silver staining (A). The arrows (lane 1) indicate the positions of molecular mass markers. In-gel assay of DNase activity of IgG_mix_ (15 µg) in a gel containing DNA before (lane 3) and after treatment with DTT (lane 4, B). DNase activity was revealed by ethidium bromide staining as a dark band on the fluorescent background. A part of the gel corresponding to lanes 3 and 4 was stained with Coomassie R250 to show the position of intact IgGs (lane 1) and separated light and heavy chains (lane 2) (B). FPLC gel filtration of one IgG_mix_ on a Superdex 200 column in an acidic buffer (pH 2.6) after Abs incubation in the same buffer (C) and its affinity chromatography on Sepharose bearing mouse IgGs against human IgGs (D): (–), absorbance at 280 nm (A_280_); (○), relative activity (RA) of IgG_mix_ in the hydrolysis of scDNA. A complete hydrolysis of 20 µg/ml scDNA for 4 h was taken for 100%. The error in the initial rate determination from two experiments in each case did not exceed 7–10%. For details, see Materials and methods.

Recently, several strict criteria have been applied to show that the DNase activity is an intrinsic property of IgGs from sera of MS patients but not from healthy donors [24]–[26]. When searching abzymes in CSF of MS patients, the IgG fraction was purified by chromatography on protein-A Sepharose in conditions to remove non-specifically bound proteins, followed by gel-filtration as in [24]–[27]. The relative amount of CSF preparations from only thirteen MS patients was enough for the purification of IgG preparations. To analyze the “average” situation for MS IgGs, we prepared a mixture of equal amounts of IgGs from sera of thirteen patients (IgG_mix_). The homogeneity of the 150 kDa IgG_mix_ was confirmed by SDS-PAGE, which showed a single band before, and two bands corresponding to the H and L chains after Ab reduction with DTT (silver staining) (Fig. 2A). To prove that DNase activity of CSF IgG_mix_ is its intrinsic property and is not due to copurifying enzymes, we applied some of well known rigid criteria [13]–[17], [33]; a) electrophoretic homogeneity of IgG_mix_ (Fig. 2A); b) gel-filtration of IgG_mix_ in conditions of “acidic shock” (pH 2.6) did not lead to the disappearance of Ab activity and the peak of activity tracked exactly with 150 kDa Abs (Fig. 2C); c) complete adsorption of the activity by Sepharose bearing monoclonal mouse Abs against human IgG light chains and its elution from the adsorbent with buffer of low pH (Fig. 2D).

In order to exclude possible artifacts due to any hypothetical traces of contaminating enzymes we analyzed additionally the DNase activity of IgG_mix_ using an *in situ* assay in SDS-PAGE gels containing DNA. After incubation, in order to allow DNase renaturation and staining with ethidium bromide, a sharp dark band at the position of DNA hydrolyzing proteins was revealed on a fluorescent background of DNA-bound ethidium bromide (Fig. 2B). After the dissociation of the IgG_mix_ using DTT, DNase activity was revealed only in the band of the separated light chains. Similar data was obtained earlier for abzymes from serum of MS patients [24]–[26].

Since SDS dissociates any protein complexes, and the electrophoretic mobility of hypothetical contaminating DNases cannot coincide at the same time with that of intact IgG and its L-chains, the detection of DNase activity in the gel region corresponding only to IgG and its light chains, together with the absence of any other bands of the activity or protein, provides direct evidence that DNase activity is an intrinsic property of MS IgG_mix_ and is not due to copurifying enzymes.

DNase activity was detected earlier in the case of IgGs from sera of 71 of 75 MS patients (∼95%) but in none of 50 healthy donors [24]–[26]. There was not any possibility to get CSF preparations from healthy donors. However, one can assume that CSF, similarly to serum preparations from healthy donors, does not contain IgGs with DNase activity.

**Figure 3:**
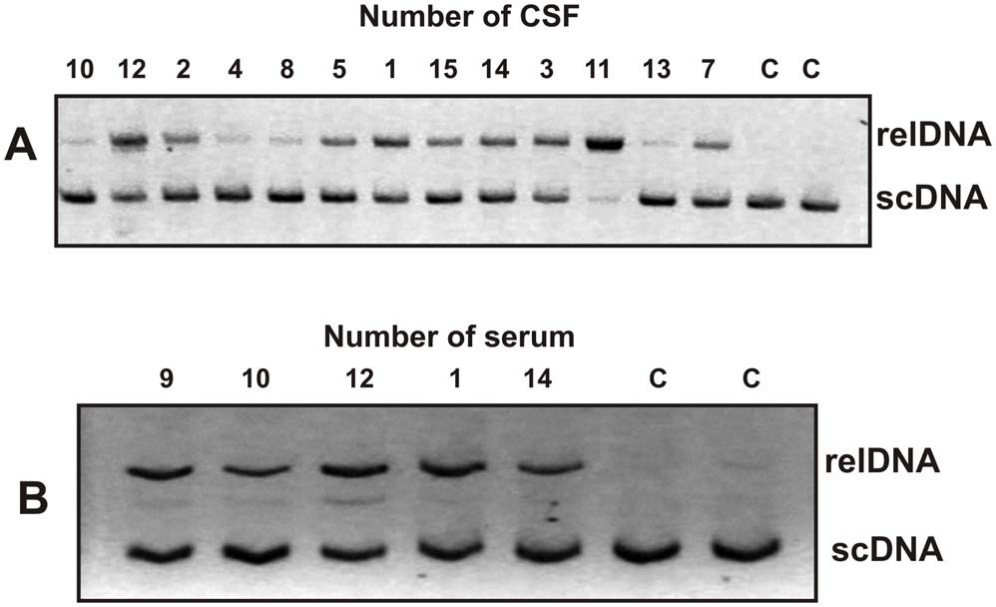
DNase activity of individual IgGs from several CSFs (A) and sera (B) of MS patients in the cleavage of scDNA. The reaction mixtures were incubated with the IgGs from CSFs (0.003 mg/ml) and sera (0.2 mg/ml) for 2 h at 37°C. Lane C in each group of IgGs corresponds to scDNA incubated without Abs mark the positions of supercoiled (scDNA) and relax DNA plasmid (relDNA) are shown.

We compared the RAs in the hydrolysis of DNA of IgG preparations from sera and CSFs of thirteen MS patients. The RAs of IgGs from sera and CSF varied markedly from patient to patient. Fig. 3 illustrates typical examples of cleavage of scDNA by IgGs (0.2 mg/ml) from the sera and CSFs (0.003 mg/ml) of several patients after 2 h of incubation at their fixed concentrations. To estimate the DNase activity quantitatively, we found the concentration for each IgG preparation corresponding to the linear part of the rate dependences on Ab concentration (the conditions of the reaction of the pseudo-first order), and the time of incubation sufficient to convert scDNA into the relaxed form without fragmentation after 1–2 h of incubation; DNA hydrolysis was in the linear range of the time courses (for example lanes 4, 8, 10, and 15 of Fig. 3A). Since all measurements (initial rates) were taken within the linear regions of the time courses and Ab concentration curves, the measured RAs for individual IgGs were normalized to standard conditions similarly to those for the preparation of total protein (Table 3).

It was surprising, but CSF IgGs possess significantly higher specific DNase activity than serum ones. Average specific DNase activity of serum IgGs (average value 11.2±4.3 pmole DNA/1 mg of Ab/1 h; range 3.6–20.8 units) is 48.5-fold lower than that for CSF Abs (average value 543.7±239.7 units; range 117.3–1190.5 units) (Table 3). At the same time, there is no good correlation between catalytic activities of IgGs from the sera and CSF, CC = +0.26 (Table 3). Interestingly, average specific RAs of serum IgGs are ∼22-fold higher than those of serum total proteins, while this difference in the case of CSF is significantly lower, only ∼5.4-fold (Table 3).

## Discussion

There are no published data concerning CSF abzymes with any catalytic activities. Data reported in this paper provide strong evidence that DNase activity is an intrinsic property of IgGs present in CSF of MS patients: it is not due to copurifying enzymes. The presence of anti-DNA Abs and DNase IgGs in CSF of MS patients is in agreement with the detection of B-cells producing anti-DNA Abs directly in active plaques and periplaque regions in MS brain and cerebrospinal fluid of a patient with MS [2]. Since sera of MS patients contain greater number of different proteins than CSFs (Fig. 1A) and the average concentration of total protein in the sera is ∼130-fold higher than in CSFs, it is not surprising that average specific RA of CSF total protein is 198-fold higher than that of the serum one. The question is why specific RAs of CSF total IgGs are significantly (48.5-fold) more active than those from sera. In addition, relative concentration of Abs (of a total pool) interacting with DNA in sera is ∼1986-fold higher than in CSF, while a difference in the concentration of total IgGs is only 198-fold, a difference of 10.3-fold (Table 2). Thus, a small specific fraction of anti-DNA Abs from the CSF may be 10-fold more active (totally ∼485-fold) than a similar small fraction of Abs interacting with DNA from the sera. In this context, some data from literature should be mentioned.

Overall, abzymes of MS patients may be significantly more active in the hydrolysis of DNA than what we have found (Table 3). As it was previously shown, the fractions of abzymes with different catalytic activities, including nuclease ones, in the serum of autoimmune patients usually do not exceed 1–7% of total immunoglobulins [13]–[17]. Since the specific activity was calculated using the total concentration of IgGs, the specific DNase activities of the individual monoclonal subfractions in a polyclonal IgG pool may be significantly higher than those of the non-fractionated IgGs. In addition, the repertoire of polyclonal Abs against different antigens in the case of sera from MS patients may be significantly wider than that of CSFs. It may be one of the possible reasons of a lower specific activity of sera IgGs.

At the same time, an ever-growing number of observations suggest that autoimmune diseases originate from defects in hematopoietic stem cells [34]. It was recently shown that the specific reorganization of immune system during the spontaneous development of a profound SLE-like pathology in MRL-lpr/lpr mice is associated with changes in the differentiation profile and the level of proliferation of bone marrow hematopoietic stem cells and with the production of DNase, ATPase, and amylase abzymes [35], [36]. Immunization of healthy mice with DNA also leads to a production of Abs with DNase activity; however, it is only associated with increased lymphocyte proliferation and suppression of apoptosis of lymphocytes in different organs (especially spleen), but not with a change in the differentiation of bone marrow cells [35], [36]. Thus, it is reasonable to suggest that B-cells of CSF of MS patients can produce not only Abs interacting with DNA [2], but also specific anti-DNA abzymes with higher DNase activity. Abzymes produced by lymphocytes against DNA in different organs of MS patients (and circulating in the blood system) may have a lower DNase activity in comparison with anti-DNA Abs of CSF or may be different ratio of abzymes and anti-DNA Abs without catalytic activity in the CSFs and sera of MS patients.

We have estimated CCs between different characteristics of CSF and sera. It was shown that there was no good correlation between several identical indexes characterizing CSFs and sera of MS patients, CCs varying in the range of −0.12 to +0.57 (Tables 2 and 3). The correlation between the total protein concentration and: a) total concentration of IgGs in CSF (CC = +0.17; columns 1 and 3) and sera (CC = +0.47; columns 2 and 4); b) relative concentration of anti-DNA Abs in CSF (CC = +0.11; columns 1 and 5) or sera (CC = −0.04; columns 2 and 6) was low (Table 2). At the same time, low but still the best positive correlation was observed between the total protein concentration and relative DNase activity of total CSF protein (CC = +0.61; columns 1 and 7), whereas in the sera these values showed negative correlation (CC = −0.49; columns 2 and 8) (Tables 2 and 3). Similar low CCs were observed for other estimated parameters. CC between the relative concentrations of IgGs and anti-DNA Abs was relatively low in CSF (CC = +0.4; columns 3 and 5), and negative in the sera (CC = −0.32; columns 4 and 6) (Table 2). Interestingly, CC between the concentration of IgGs and the relative specific DNase activity of CSF Abs (CC = +0.16; columns 3 and 11) was lower than that of the sera (CC = −0.58; columns 4 and 12) (Tables 1 and 2). Finally, the relative concentration of total anti-DNA Abs correlated with the relative specific IgG DNase activity better in the sera (CC = +0.51; columns 6 and 12) than in CSF (CC = +0.11; columns 5 and 11) (Tables 2 and 3). An additional question is why there is no good correlation between various indexes, characterizing different MS patients.

An analysis of correlation between titers of Abs to DNA as well as to MBP and 13 different standard clinical parameters including Poser criteria (indexes for evaluation of damage to functional systems: pyramidal functions; cerebellar functions; functions of brain stem; sensitive functions; functions of intestines and urinary bladder; visual functions; cerebral (psychical) functions and sum of these characteristics) in the case of 49 patients with MS was carried out [17]. For the whole group of MS patients, the absolute values of positive CCs between titers of anti-DNA or anti-MBP Abs and clinical Poser indexes were very low (between 0.01 and 0.19), absent (∼0), or even negative (−0.02 to −0.07) and statistically non-significant. Several CCs became higher and reached values up to 0.1 to 0.55 and −0.04 to −0.47 after the division of cohort into subgroups of patients with primary progressing, secondary progressing and remitting course of the disease [17].

The groups of primary progressing remitting course and secondary progressing course of MS patients were not “homogenous” with respect to the patients’ characteristics, and their further subdivision using cluster and factorial analysis revealed high statistically significant correlation coefficients [17]. For example, for one sub-subgroup of the remitting course subgroup, a direct dependence between titers of anti-MBP and symptoms of lesions of the pyramidal tract was observed (CC = 0.92). In some cases, correlations of the opposite sign were observed for the same pairs of analyzed parameters for the three subgroups with different MS courses and their sub-subgroups obtained by cluster analysis from the subgroups.

The absence of a definite dependence between titers of anti-DNA and anti-MBP Abs and these parameters with standard clinical indices may be due to several reasons. MS is an extremely multifactorial disease, in which similar pathomorphological and clinical indices manifested as MS may result from very different underlying processes and conditions [37], [38]. For example, in each MS patient, the “relative stability” of different organs and their functions to the destructive effect of transient immune system errors can be significantly different depending on the genetic background and environmental stress factors, including geographic ones [37]–[39]. Some proteins of influenza, herpes, polyoma, Epstein–Barr and other viruses and of some bacteria have been reported to mimic human myelin proteins, and these infections can therefore lead to immunization with their proteins and stimulate the subsequent formation of Abs to myelin and finally to the development of autoimmune reactions [38], [40]–[43]. In individual MS patients, the development of autoimmune reactions can be stimulated by different viral or bacterial infections as well as various toxic chemicals. Furthermore, it should also be taken into account that MS is pathology of at least two- phases [44]. The cascade of reactions corresponding to the first inflammatory phase is very complicated and involves many proteins, enzymes, cytokines, and chemokines inducing macrophages and other cells producing NO^•^ radicals and osteopathin [38], [44]. The complex and coordinated action of T- and B-cells, complement system, inflammation mediators, and auto-Abs result in the formation of demyelinization nodi and interruption of axon conductivity. The neurodegenerative phase of MS that ensues later is directly connected with the neural tissue destruction in these patients [38], [44]. Therefore, any analysis of biochemical, immunological and clinical indices must take into account of the current stage of the disease. Obviously, quite different characteristics of pathologic processes can be obtained in individual patients as the disease progresses against the background of the continually changing immunoregulation, including exhaustion of different compensatory and adaptive mechanisms and systemic metabolic changes. This makes the clinical course of MS hardly predictable in individual patients [38], [44]. Therefore, it is not surprising that we could not find a high statistically significant correlation of titers of Abs to DNA and RAs of abzymes with all parameters measured, since each patient can be characterized by an individual combination of genetic, environmental, chronic, inflammatory, autoimmune, demyelinating, neurodegenerative and other factors.

Overall, all data obtained demonstrate that the DNase activity is an intrinsic property of IgGs deriving from CSF and sera of MS patients. These IgGs are polyclonal and may consist of extremely different repertoires of DNase subfractions in the case of CSF and sera. We have shown previously that the appearance of abzymes specifically hydrolyzing DNA is among the earliest and clear signs of autoimmune reactions in a number of autoimmune diseases when titres of Abs to DNA or other auto-antigens have not yet increased significantly and correspond to their ranges for healthy donors [16], [17], [24]–[26]. Therefore, detection of DNase Abs in the sera and CSF of peoples can be considered as an additional index for early diagnostic of this pathology.

## Materials and Methods

### Chemicals and Donors

Most chemicals, proteins, Protein G-Sepharose, and the Superdex 200 HR 10/30 column were from Sigma or GE Healthcare. Fifteen consecutive MS patients (11 women and 4 men; mean age = 39±12.5 years) satisfying the criteria for definite MS according to the classification of McDonald [29] and admitted to the Multiple Sclerosis Center of the University of Ferrara during the period from January 2012 to October 2012 were retrospectively selected for the study. Disease severity was scored in all MS patients at the time of sample collection using Kurtzke’s Expanded Disability Status Scale (EDSS) [30] (mean at entry = 1.8±1.4; range from 0 to 4.0). Clinical course (RR and PP), clinical activity (relapse at time of sampling), and MRI activity (the presence of gadolinium enhancing lesions at MRI examination) were analyzed as described previously [32].

At entry none of the patients had fever or other symptoms or signs of acute infections. Moreover, at the time of sample collection none of the patients had received any potential disease-modifying therapies during the 6 months before the study.

### Sample Preparation

The blood and CSF sampling protocols confirmed the local committee for medical ethics in research (Comitato Etico della Provincia di Ferrara) that approved our study in accordance with Helsinki ethics committee guidelines including written consent of patients confined to present of their blood and CSF for diagnostics of a possible disease and scientific purposes. The protocol was approved at 31 May 2007 and it was focused on the creation of a biological bank of CSF and serum samples, and related clinical data of patients with MS and other neurological diseases including: a) a study of potential markers (especially proteins) for diagnostic and prognostic significance in diseases of the nervous system; b) specific antibodies directed against antigens potential exogenous and/or endogenous; c) presence of pathogens (mostly viruses or bacteria) for association studies and pathogenesis; d) neurotransmitters and their metabolites; e) a study of different properties of different markers.

CSF and serum samples were collected under sterile conditions and stored in aliquots at 80°C until assay. “Cell-free” CSF samples were obtained after centrifugation at room temperature of specimens taken by atraumatic lumbar puncture performed for purposes of diagnosis in the absence of contraindications. Serum samples derived from centrifugation of blood specimens withdrawn by puncture of an anterocubital vein at the same time of CSF extraction. Paired CSF and serum samples from MS patients were stored and measured under exactly the same conditions. Informed consent was given by all patients before inclusion and the study design was approved by the Regional Committee for Medical Ethics in Research. CSF and serum IgG levels were measured by immunochemical nephelometry with the Beckman Immage 800 Immunochemistry System (Beckman Instruments, Inc. Fullerton, CA. USA) according to the procedure of Salden et al. [45].

### Analysis of Protein Concentrations

In all cases, protein concentration in the intact CSF, sera of MS patients and final solutions of Abs was measured using Bradford assay with a bovine serum albumin standard. The concentration of IgGs after their purification by affinity chromatography on protein G-Sepharose (see below) was measured in the same way.

### Analysis of IgG and anti-DNA Abs Concentration

Relative concentrations of IgGs in the intact CSF and the sera of MS patients were analyzed using special quantitative isoelectrofocusing and immunoblotting test system in Italy according to the standard manufacturer’s protocol and equipment (IgG IEF, Helena Laboratories, Gateshead, Tyne and Wear, UK). In addition, the relative concentrations of IgGs in the intact CSFs and the sera of MS patients were measured after Abs purification by affinity chromatography on protein G-Sepharose (see below).

The titers of anti-DNA Abs were determined using standard assay plates with immobilized double-stranded DNA, horseradish peroxidase-conjugated mouse Abs against human IgG, and tetraethyl benzidine as substrate according to the standard manufacturer’s protocol (Vector, Russia). The preparations of human blood serum and CSF were diluted respectively 1000 and 2 times and 100 µl of final solution was added to the strips. The reaction was stopped with sulphuric acid and optical density (A_450_) of the solutions was determined using a Uniskan II plate reader (MTX Lab Systems, USA). The relative concentration of anti-DNA Abs in the samples was expressed as the difference in the relative absorbance at 450 nm (average of three measurements) between the experimental samples and the control samples containing no Abs. As additional controls, we have used preparations complete devoid of Abs after passage of CSFs through protein G-Sepharose and protein A-Sepharose. There was no difference in the relative absorbance at 450 nm of CSF preparation containing no Abs and controls containing a buffer only. Finally, A_450_ values were recalculated on respective biological fluids without dilution.

### IgG Purification

Electrophoretically and immunologically homogeneous IgGs were obtained by sequential affinity chromatography of the CSF and serum proteins on protein G-Sepharose and FPLC gel filtration similarly to [24]–[27]. In each case the protein corresponding to the central part of IgG peaks was concentrated and used in further purification or analysis.

IgGs from CSF were incubated in 50 mM glycine-HCl (pH 2.6) containing 0.2 M NaCl for 20 min at 25°C. Separation of the IgGs under “acid shock” conditions was done by FPLC gel filtration on a Superdex 200 HR 10/30 column equilibrated with 50 mM glycine-HCl (pH 2.6) containing 0.1 M NaCl as previously described [24]–[27]. After 1–2 weeks of storage at 4°C in order to refold after the acid shock, the Abs were used in the activity assays described below.

In some cases, electrophoretically homogeneous IgGs were chromatographed on Sepharose bearing immobilized monoclonal mouse Abs against light chains of human IgGs as in [24]–[27]. The protein was applied to the column (1 ml) equilibrated with 20 mM Tris-HCl (pH 7.5) containing 0.1 M NaCl and the column was washed with the same buffer containing 0.3 M NaCl. Abs were eluted in 0.05 M glycine-HCl (pH 2.6), neutralized, dialyzed and sterilized as described above.

### DNase Activity Assay

DNA-hydrolyzing activity of total protein and IgG preparations was analyzed using supercoiled (sc)DNA as earlier described for the analysis of DNase I and human serum catalytic antibodies [24]–[26]. The reaction mixture (20 µl) contained 20 µg/ml scDNA pBluescript, 5 mM MgCl_2_, 1 mM EDTA, 20 mM Tris-HCl (pH 7.5), and 0.003–0.2 mg/ml Abs or initial preparations of the sera or CSF (total protein) finally diluted respectively 1000- and 15-fold. Reaction mixtures were incubated for 0.5–3 h (standard time, 2 h) at 37°C. The cleavage products were analyzed by electrophoresis in 1% agarose gel. The images of ethidium bromide-stained gels were captured on a Sony DSC-F717 camera and a relative amount of DNA in different bands was analyzed using ImageQuant v5.2 (Molecular Dynamics). The activities of IgG preparations were determined as a decrease in the percentage of DNA converted from the initial supercoiled form to the relaxed form, corrected for the distribution of DNA between these bands in the control (incubation of pBluescript in the absence of Abs). All measurements (initial rates) were taken within the linear regions of the time courses (15–40% of DNA hydrolysis) and then recalculated to the standard conditions (see Tables).

### SDS-PAGE Assay of DNase Activity

SDS-PAGE analysis of Abs for homogeneity and for the polypeptide spectrum of the sera and CSF was performed in a 5–16% gradient gel containing 0.1% SDS (Laemmli system) as described in [24]–[26]. IgGs were used before and after their treatment with 10 mM dithiothreitol. The polypeptides were visualized by silver and Coomassie Blue staining [24]–[26].

*In situ,* experiments DNase activity of IgGs after SDS-PAGE was analyzed in gel containing calf thymus DNA (5 µg/ml) under non-reducing conditions as in [24], [25]. Before the electrophoresis, IgG samples were incubated at 22°C for 10–20 min in 20 mM Tris-HCl (pH 7.5) containing 0.1% SDS. To restore the enzymatic activity after SDS-PAGE, SDS was removed by incubating the gel for 1 h at 22°C in 20 mM Tris-HCl (pH 7.5) and washing the gel five times with the same buffer. To refold the protein after SDS treatment and to assay it for DNase activity, longitudinal slices of the gel were incubated at 25°C for 15–48 h in the reaction buffer containing 20 mM Tris-HCl (pH 7.5), 4 mM MgCl_2_, and 0.2 mM CaCl_2_. To visualize the products of DNA hydrolysis, the gel was stained with ethidium bromide. The same ethidium bromide-stained or parallel longitudinal slices were used to detect the position of IgG in the gel by Coomassie Blue staining.

### Statistical Analysis

The results are reported as mean ± S.E. of at least three independent experiments for each sample analyzed. Errors in the values were within 5–7%. The correlation coefficients (CC) between sets of different samples were analyzed.
